# *Zfp521* promotes B-cell viability and *cyclin D1* gene expression in a B cell culture system

**DOI:** 10.1016/j.leukres.2016.03.013

**Published:** 2016-07

**Authors:** Salma Al Dallal, Kathryn Wolton, Kathryn E. Hentges

**Affiliations:** Faculty of Life Sciences, University of Manchester, Manchester M13 9PT, UK

**Keywords:** *Zfp521*, *Pax5*, *Cyclin D1*, *Zfp423*, *Ebf1*, *Evi3*

## Abstract

Leukemia arises due to the dysregulated proliferation of hematopoietic progenitor cells. Errors in the multi-step commitment process result in excessive numbers of immature lymphocytes, causing malignant disease. Genes involved in the differentiation of lymphocytes are often associated with leukemia. One such gene, *Zfp521*, has been found to cause B-cell leukemia in mice when over-expressed. The role of *Zfp521* in B-cell differentiation, and the mechanisms by which it leads to leukemic transformation, are unclear. In this study we report that *Zfp521* knockdown causes apoptosis in a B-cell culture system and promotes down-regulation of genes acting at late stages of B-cell differentiation. We identify *Pax5* and *cyclin D1* as *Zfp521* target genes, and suggest that excessive B-cell proliferation observed in mice with retroviral insertions near the *Zfp521* gene is due to an up-regulation of *cyclin D1* in B-cells. Overall, these results suggest links between dysregulated *Zfp521* and B-cell survival.

## Highlights

• Knockdown of *Zfp521* in BCL1 cell culture reduces viability and promotes apoptosis.
• Genes expressed in B cells are down-regulated in cells with *Zfp521* knockdown.
• *Cyclin D1* expression is increased in mouse tumors with *Zfp521* over-expression.

## 1 Introduction

Leukemia originates due to errors in the hematopoietic differentiation of stem cells into mature lymphocytes [1]. Lymphocyte development requires a multi-step process whereby common lymphoid progenitors differentiate into B and T lineage-specific cells [2]. Developmental control of early B lineage cell differentiation is exerted by a regulatory network of key transcription factors [3], [4]. Sequential actions of these transcription factors results in the multi-step differentiation of mature B-cells from immature progenitors in the bone marrow [4].

Recombinant inbred (RI) mouse stains are a significant resource for leukemia gene discovery [5]. These mice harbor somatic viral insertions that alter the expression of tumor suppressor genes and proto-oncogenes [5], [6]. Sites of proviral insertion that are common to many animals within a strain or between multiple strains identify genomic locations where proto-oncogenes or tumor suppressor genes reside. The RI strain AKXD-27 possessed B-lineage lymphomas with a common proviral insertion site, designated as ecotropic viral integration site 3 (*Evi3*) [7]. Within the AKXD-27 strain 70% of mice exhibiting pre-B cell and B-cell tumors contained proviral insertions at the *Evi3* locus; no rearrangements at the *Evi3* locus were found in either T-cell or myeloid tumors [7], [8]. The high frequency of B-lineage lymphoma in mice with a proviral insertion at the *Evi3* locus suggests that proviral insertion alters the expression of genes near the insertion site to promote B-lineage lymphoma.

Analysis of the genomic region surrounding the *Evi3* retroviral integration site revealed that the virus had inserted upstream of a previously uncharacterized gene, which encodes a 30-zinc-finger protein with predicted DNA-binding and protein interaction domains [8], [9]. This gene was found to be up-regulated in tumors with retroviral insertions at Evi3, due to the strong viral promoters driving endogenous gene expression [9]. The human ortholog of this gene, *EHZF*, was identified due to its specific expression in CD34^+^ early hematopoietic progenitor stem cells [10]. Due to its zinc finger motifs, the gene at the *Evi3* insertion site has been renamed *Zfp521* in mice and *ZNF521* in humans.

Although studies on the molecular function of *Zfp521* have revealed a role in transcriptional regulation via chromatin remodeling [11], its place within the transcriptional network regulating B-cell differentiation remains unclear. To better understand the role of *Zfp521* in B-lymphocytes, we developed a knockdown system in the lymphoblast cell line BCL1, which secretes IgD and IgM antibodies [12]. We assayed B-cell gene expression in this system, and found that certain genes which were up-regulated in B-cell tumors from AKXD-27 mice with *Evi3* retroviral insertions [8] were conversely down-regulated in *Zfp521* knockdown cells. Knocking down *Zfp521* resulted in decreased cellular viability and increased cellular apoptosis. Using a cell viability rescue assay, we identified *cyclin D1* as a potential mediator of increased B-cell proliferation in *Zfp521* over-expressing leukemias, and propose a position for *Zfp521* within the B-cell differentiation transcriptional regulatory network.

## 2 Materials & methods

### 2.1 Cell culture

Mouse lymphoblast cells (BCL1; ATCC^®^ TIB-197) were cultured in RPMI 1640 medium supplemented with 2 mM L-glutamine (Lonza), 0.05 mM 2-mercaptoethanol (Sigma-Aldrich), 15% FBS, 5% penicillin, 5% streptomycin at 37 °C with 5% CO_2_.

### 2.2 *Zfp521* shRNA constructs

shRNA constructs were purchased from OriGene (OriGene Technologies: SR422637). Four independent *Zfp521* shRNA expression vectors with a CMV-Green Fluorescent Protein (GFP) marker were combined at equal concentration for transfection. A vector containing scrambled *Zfp521* shRNA sequence and an empty vector lacking any shRNA sequence were used as controls.

### 2.3 Transfection

1 μg plasmid DNA was transfected into 1 × 10^5^ BCL1 cells with FuGENE HD (Roche) in OptiMEM Media (Sigma). Transfection efficiency was calculated based on the GFP expression for each individual plasmid.

### 2.4 Viability assay

BCL1 were plated in triplicate at a density of 1 × 10^5^ cells per well in 96-well plate. After 24 h, cells were transfected with shRNA plasmids or appropriate control plasmids and cultured for 1, 3 or 7 days. Cultured cells were incubated with CellTiter 96^R^ Aqueous Non-Radioactive Cell Proliferation Assay (MTS) according to manufacturer’s instructions (Promega; no. G5421). Absorbance was recorded at 490 nm (Bio-Tek Powerwave HT Microplate Reader). Each assay was repeated with six technical replicates and three biological replicates.

### 2.5 Trypan blue stain

BCL1 cells were trypsinized in 1 ml trypsin (Sigma); cells were re-suspended in BCL1 media. An equal amount of cell suspension and trypan-blue solution (Sigma) were mixed together. Cells were visualized under light microscopy. Five different squares from a hemocytometer grid were counted to determine the total cell number and number of dead cells (stained blue). Each assay was repeated with three technical replicates and three biological replicates.

### 2.6 Caspase-3/7 assay

BCL1 cells were plated in triplicate at a density of 1 × 10^5^ cells per well in 96-well plate. After 24 h, cells were transfected with *Zfp521* shRNA or control plasmids as described. Caspase activity was assessed using Apo-ONE Homogenous Caspase-3/7 Assay (Promega; no: G7792) according to the manufacturer’s instructions. Absorbance was recorded at 490 nm. Wells with no cells were used a blank, and the average absorbance value of the blank was subtracted from the average absorbance value for each treatment condition. Fold change for each experimental condition was calculated by normalization to mock-transfected cells. Each assay was repeated with three technical replicates and three biological replicates.

### 2.7 Real-time quantitative PCR analysis

Total RNA was extracted with TRI reagent (Sigma), treated with DNaseI (Promega), reverse transcribed using random primers and prepared for real-time quantitative PCR (Promega GoTaq qPCR mix) according to manufacturer’s instructions. Primer sequences are listed in Supplemental Table 1. Reactions were performed in triplicate. Thermal cycling parameters were: 95 °C for 10 min, 40 cycles of 95^ °^C for 15 s, and 60^ °^C for 1 min. Expression levels were normalized to *18S RNA*. Expression was analysed using the ΔΔCT method [13]. Results were analyzed by *t* test for statistical significance.

### 2.8 Site-directed mutagenesis

Site-directed mutagenesis primers were designed using software from New England BioLabs (http://www.neb.com/products/e0554-q5-site-directed-mutagenesis-kit). The Q5 site-directed mutagenesis kit (New England BioLabs; E0554S) was used following manufacturer’s instructions to create a stop codon within the *Zfp521* sequence.

Forward primer: 5′-TGCACAGCTGAGACAGCTGCC-3′

Reverse primer: 5′-CAGCGTCCTCCTCCAACTC-3′

### 2.9 Rescue assay

BCL1 cells were plated in 24-well plates at a density of 1.0 × 10^6^ cells per well. Cells were transfected with *Zfp521* shRNA plasmid or control plasmids. The plates were incubated for 3 days, after which cells were transfected again with the plasmids listed below. An identical replicate control plate was mock-transfected without any addition of any plasmids. Plasmids transfected into knockdown cells: full-length *Zfp521*, mutant *ΔZfp521*, *Zfp521Stop*, *Pax5* wild type, *Pax5* V26G, *Ebf1*, and *cyclin D1*. Following the rescue transfection cells were subjected to the viability assay described above. The ratio of viable cells at day 7 post-transfection for rescued cells to cells without rescue was calculated for each transfection condition. A pairwise *t* test was performed to assess statistical significance.

## 3 Results

### 3.1 Generation of a *Zfp521* B-cell knockdown system

**Fig. 1:**
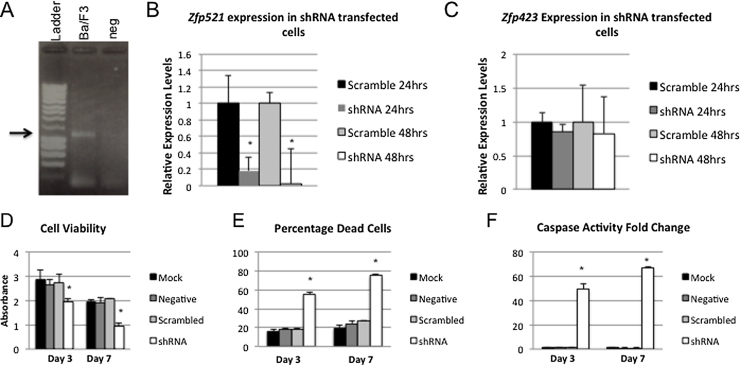
The *Zfp521* BCL1 B-cell knockdown system. A. *Zfp521* expression is present in the Pro-B cell line Ba/F3. Ladder = Bioline Hyperladder I, neg = no template control. B. *Zfp521* expression is significantly reduced in BCL1 cells transfected with a specific shRNA as compared to mock-transfected cells at both 24 h (*p = 0.03; *t* test) and 48 h (*p = 0.006; *t* test). C. *Zfp423* expression is unaffected in cells transfected with *Zfp521* shRNA at either 24 h (p = 0.19; *t* test) or 48 h (p = 0.69; *t* test). D. The presence of viable cells is significantly reduced in cultures transfected with *Zfp521* shRNA when compared to cells undergoing mock transfection at 3 days and 7 days post-transfection (*p < 0.05; *t* test), or transfection with vector containing no shRNA (negative) or scrambled shRNA sequence. E. The percentage of dead cells at days 3 and 7 post-transfection as identified by trypan blue staining is significantly increased in cells transfected with *Zfp521* shRNA as compared to cells undergoing mock transfection (*p < 0.05; *t* test), or transfection with vector containing no shRNA (negative) or scrambled shRNA sequence. F. The fold-change in the levels of Caspase 3/7 activity in cells transfected with *Zfp521* shRNA or control plasmids as compared to mock-transfected cells. A significant increase in Caspase 3/7 activity is detected in *Zfp521* shRNA transfected cells compared to mock-transfected cells at day 3 and day 7 (*p < 0.05; *t* test) post-transfection.

The majority of tumors found in AKXD-27 mice with over-expression of *Zfp521* were of the B-lineage, at the Pro-B cell stage of differentiation [8]. *Zfp521* expression has been reported in wild type mouse B-cells during early stages of B-cell differentiation [14], and we have detected *Zfp521* expression in the IL-3 dependent murine pro-B-cell line Ba/F3 [15] (Fig. 1A), as well as in the mouse lymphoblast cell line BCL1 (Fig. 1B). To investigate the effects of reduced *Zfp521* expression in B-cells, BCL1 cells were transfected with either a cocktail of four *Zfp521* shRNA knockdown plasmids, a gene-specific scrambled control plasmid (scrambled *Zfp521* target sequence), or an empty vector control. Mock-transfected cell were also analyzed as a further negative control. *Zfp521* expression was assayed by qPCR in BCL1 cells 24 h and 48 h post-transfection. There was a significant decrease in *Zfp521* expression in cells transfected with the knockdown plasmid cocktail as compared to cells transfected with the scrambled control plasmid at both 24 and 48 h post-transfection (Fig. 1B). We sought to confirm that our BCL1 cells demonstrated a specific knockdown of *Zfp521* without affecting expression of its paralog *Zfp423;* therefore we measured the expression levels of *Zfp423* in cells transfected with *Zfp521* shRNA or the scrambled control plasmid. We found no difference in the expression levels of *Zfp423* in cells with *Zfp521* knockdown as compared to control plasmid transfected cells, confirming that the knockdown is specific to *Zfp521* (Fig. 1C).

### 3.2 *Zfp521* knockdown cell phenotype

We employed an enzymatic cell viability assay to determine the number of live cells in our different transfection conditions. We found that *Zfp521* knockdown cells had reduced viability as compared to other transfection conditions, with a significant decrease in cell viability by day 7 post-transfection (Fig. 1D). To confirm these findings, trypan blue staining was used to identify dead cells in each transfection condition. The percentage of dead cells was significantly increased in the *Zfp521* knockdown transfection cells as compared to control transfection samples (Fig. 1E).

Because we detected reduced cell viability in *Zfp521* knockdown cells, we sought to determine if the reduced viability was due to increased apoptosis. We measured caspase-3/7 activity at 24 h, 3 days, and 7 days post-transfection in *Zfp521* knockdown and control plasmid transfected cells. The fold change in levels of apoptotic cells was increased in cells transfected with the *Zfp521* shRNA plasmid cocktail as compared to mock transfection conditions, with a significant difference detected at day 7 post-transfection (Fig. 1F).

### 3.3 B-cell gene expression analysis

**Fig. 2:**
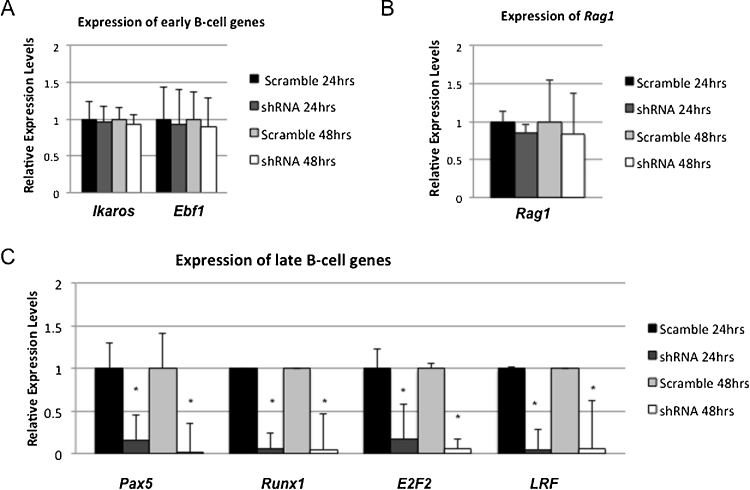
Expression of B-cell genes in *Zfp521* knock-down cells. A. The expression of early B-cell genes was assayed by qPCR at 24 and 48 h post-transfection in BCL1 cells transfected with *Zfp521* shRNA or scrambled control plasmid. B. The expression of the recombination gene *Rag1* in scrambled control or *Zfp521* shRNA transfections. C. The expression of late B-cell marker genes at 24 and 48 h post-transfection. Expression was significantly reduced in *Zfp521* shRNA transfected cells as compared to cells transfected with scrambled plasmid for all genes reported in Panel D (*p < 0.05; *t* test). All panels show expression normalized to *18S* RNA control.

To determine the effect of the knockdown of *Zfp521* on genes required for B-cell differentiation, we examined gene expression using quantitative RT-PCR in cells with *Zfp521* knockdown as compared to control transfected cells. We analyzed the expression of B-cell transcription factors required prior to the Pro-B-cell stage of differentiation: *Ikaros* and *Ebf1* [4]. *Ikaros* plays a critical role in regulating lymphocyte development, function, and homeostasis [16]. Early B-cell factor (*EBF*) is a transcription factor involved in the transcriptional regulation of many B-cell restricted genes, which is essential for B lymphocyte development [17]. There was no reduction in the expression of either gene in *Zfp521* knockdown cells (Fig. 2A). *Rag1* and *Rag2* are lymphocyte-specific genes involved in V(D)J rearrangement of immunoglobulin (Ig) [18], which are expressed in early lymphoid progenitors [19]. No difference was observed in the expression of *Rag1* in cells with *Zfp521* knockdown as compared to control-transfected cells (Fig. 2B). We analyzed the expression of genes acting at later stages of B-cell differentiation: *Pax5*, *Runx1*, *E2F2*, and *Lrf*. *Pax5* is expressed from early pro-B stage until the final stage of differentiation [20], and functions at the Pro-B-cell stage to activate genes crucial to B-cell lineage differentiation and repress genes required for commitment to other hematopoietic lineages [21]. *Runx1* is expressed in early B-cell progenitor and in immature and mature B-cells [22]. Mice with deletions of *E2F1* and *E2F2* have inhibited B-cell differentiation, with development arrested at the pre-BII stage, indicating a requirement for E2F2 at the Large Pre-BII to Small Pre-BII differentiation transition [23]. *Lrf* regulates the lineage fate of mature B cells, and is highly expressed in lymphoma cell lines [24]. We found reduced *Pax5*, *Runx1*, *E2F2*, and *Lrf* gene expression levels in *Zfp521* knockdown cells as compared to controls (Fig. 2C).

### 3.4 *Zfp521* knockdown rescue assay

**Fig. 3:**
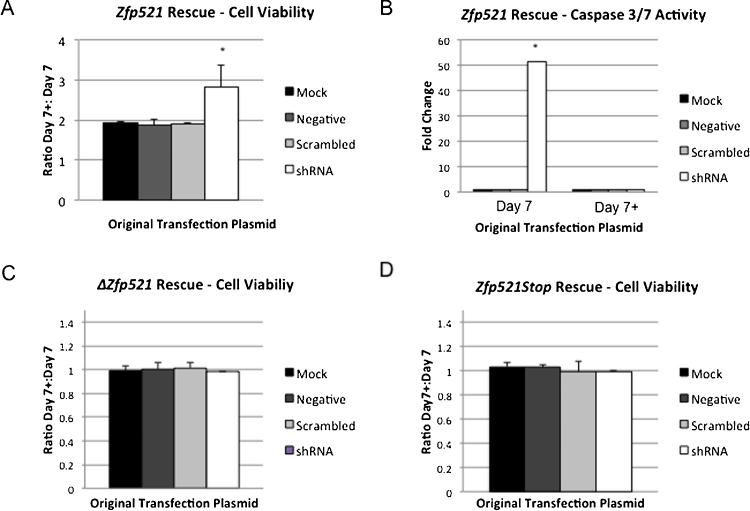
Viability and apoptosis levels in rescue assay cell cultures. A. The ratio of cell viability measured on day 7 post-transfection for cells with *Zfp521* wild type rescue plasmid added (day 7 + ) to cells without rescue plasmid added (day 7). A significant increase in viability was found in shRNA cells with *Zfp521* wild type rescue plasmid added as compared to mock-transfected cells with rescue (*p < 0.05; *t* test). B. The fold-change in Caspase 3/7 activity in each condition as compared to mock-transfected cells. Cells without rescue are shown on the left, and cells with rescue plasmid added are shown in the right. A significant different was found between caspase activity levels in cells without *Zfp521* rescue plasmid transfection to cells with rescue (*p < 0.05; *t* test). C. The ratio of cell viability measured on day 7 post-transfection for cells with *ΔZfp521* plasmid added. No significant difference was detected in shRNA cells with *ΔZfp521* plasmid added as compared to mock-transfected cells (p = 0.67; *t* test). D. The ratio of cell viability measured on day 7 post-transfection for cells with *Zfp521Stop* plasmid added. No significant difference was detected in shRNA cells with *Zfp521Stop* plasmid added as compared to mock-transfected cells (p = 0.15; *t* test). For all panels, the original transfection condition is shown in the legend.

To confirm that the reduced cell viability and increased apoptosis we saw in BCL1 knockdown cells was due to a loss of *Zfp521* expression, we developed a rescue assay system in these cells. Cells were transfected with the *Zfp521* knockdown shRNA cocktail or control plasmids as described above. On day 3 after the shRNA transfection, cells were transfected with a rescue construct. Cell viability was measured on day 7 after the rescue transfection. The ratio of viable cell number between cells receiving the rescue plasmid (denoted 7 + ) compared to control cells receiving no rescue plasmid (denoted 7) was calculated. We found that the addition of wild type *Zfp521* resulted in a rescue of cell viability in cells originally transfected with the *Zfp521* shRNA (Fig. 3A). The presence of caspase 3/7 activity was also measured at day 7 after the recue transfection. Cells that were not transfected with the *Zfp521* rescue plasmid showed a significant increase in apoptosis following *Zfp521* knockdown, however no such increase was noted in cells with *Zfp521* rescue transfection (Fig. 3B).

Prior studies have shown that the final 4 zinc fingers of Zfp521 are required for protein-protein interactions with the B-cell transcription factor EBF1 [25]. A mammalian expression construct lacking the final 6 zinc fingers of Zfp521 has previously been demonstrated to modulate EBF1 activity [8]. This construct (Δ*Zfp521*) did not rescue cell viability in cells transfected with the *Zfp521* shRNA (Fig. 3C). The Δ*Zfp521* construct was generated through an internal restriction enzyme deletion of the wild type *Zfp521* expression plasmid, and therfore has a reduced nucleotide length compared to the wild type *Zfp521* expression plasmid. To confirm that the lack of rescue in *Zfp521* knockdown cells was not an artifact of the Δ*Zfp521* construct, we generated another mutant *Zfp521* expression construct through site-directed mutagenesis. This new construct (*Zfp521Stop*) is the same as the wild type *Zfp521* plasmid with the exception of a single nucleotide substitution that generates a premature stop codon at amino acid 58 of Zfp521. The *Zfp521Stop* construct was also unable to rescue cell viability in our assay (Fig. 3D).

### 3.5 *Zfp521* target gene rescue assay

**Fig. 4:**
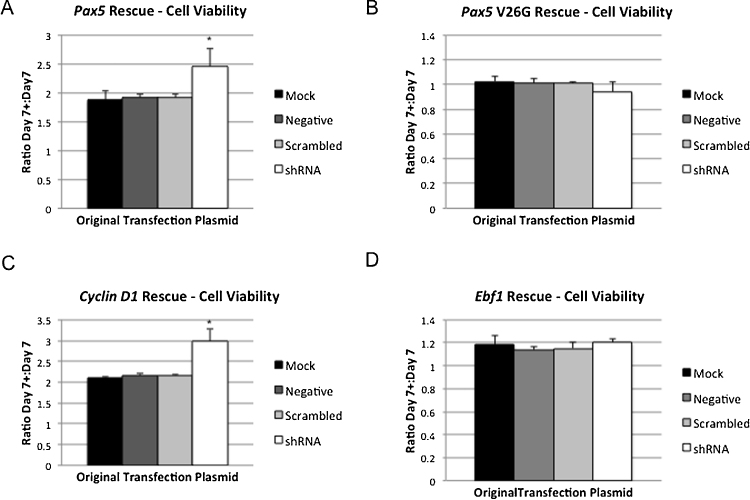
Cell viability measurements in rescue assays. A. The ratio of viable cells detected in cultures at day 7 post-transfection with wild type *Pax5* rescue plasmid (7+) compared to cells mock transfected on day 7. A significant increase in viable cell number is seen in cells originally transfected with *Zfp521* shRNA when compared to other transfection conditions (*p < 0.05; *t* test). B. The ratio of viable cells at day 7 post-transfection with Pax5 V26G mutant construct compared to cells without rescue. No significant differences were detected between any transfection groups. C. The ratio of viable cells in rescue assays with the addition of *cyclin D1* plasmid as compared to cells with no rescue. A significant increase in viable cell number is seen in cells originally transfected with *Zfp521* shRNA when compared to other transfection conditions (*p < 0.05; *t* test). D. The ratio of viable cells at day 7 post-transfection with *Ebf1* plasmid compared to cells without rescue. No significant differences were detected between any transfection groups. For all panels, the original transfection condition is shown in the legend.

We used our rescue assay system to examine potential downstream targets of Zfp521. We examined *Pax5* because *Pax5* expression levels are increased in lymphoid tumors from mice with retroviral insertions at the *Evi3* site [8], supporting the hypothesis that *Pax5* is downstream of *Zfp521*. To control for changes in viable cell number caused by general proliferative actions of the rescue candidates, we calculated the ratio of increase in viable cell number on day 7 for cells in each knockdown condition receiving the rescue plasmid (day 7 + ) as compared to cells without the addition of rescue plasmid (day 7). We found that the addition of wild type *Pax5* resulted in a significant increase in cell viability in *Zfp521* knockdown cells (Fig. 4A). To determine whether the DNA binding function of Pax5 was required for rescue, a construct containing a V26G mutation in Pax5 that lacks DNA binding ability was used [26]. The addition of mutant *Pax5* showed no increase in viability of *Zfp521* knockdown cells (Fig. 4B).

Notably, *cyclin D1* has been identified as an indirect target of *Zfp521* in chondrocytes, and depletion of *Zfp521* in chondrocytes results in reduced cell proliferation [27]. We wished to examine whether the cell proliferation defects observed in *Zfp521* knockdown cells might therefore be a consequence of perturbed *cyclin D1* activity. Our rescue assay confirmed that the addition of *cyclin D1* rescued cell viability specifically in the *Zfp521* knockdown cells (Fig. 4C), suggesting that loss of *Zfp521* disrupts *cyclin D1* regulation in B-cells.

The B-cell transcription factor *Ebf1* was found to be over-expressed in tumors with *Zfp521* retroviral insertions [8], and is a putative binding partner for Zfp521 [28], [29]. Yet in our knockdown assay we found no significant alterations in *Ebf1* expression levels. To further delineate the relationship between *Ebf1* and *Zfp521* we examined whether the introduction of *Ebf1* expression would affect *Zfp521* knockdown cell viability in our rescue assay. However, the addition of *Ebf1* did not rescue cell viability defects (Fig. 4D), consistent with the finding that *Ebf1* expression is not altered by the knockdown of *Zfp521*. This finding suggests that *Ebf1* is not a downstream target of Zfp521.

### 3.6 AKXD27 tumor expression analysis

**Fig. 5:**
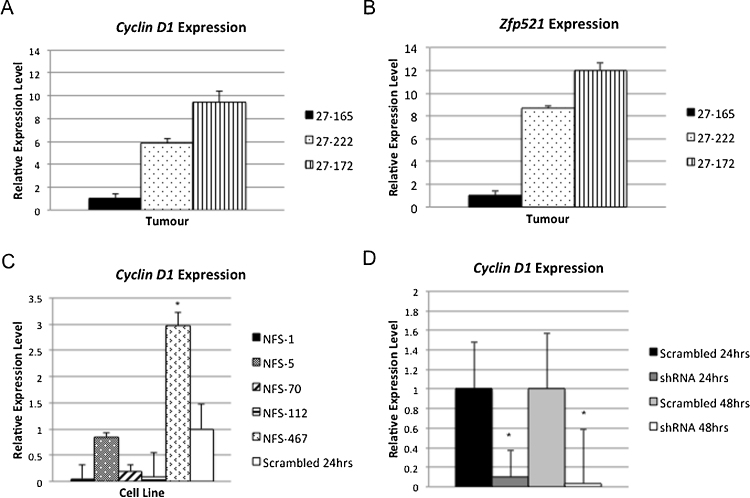
Expression analysis in AKXD27 tumors and leukemia cell lines. A. *cyclin D1* expression measured by qPCR in tumors from mice with retroviral insertions at *Evi3* (27-222 and 27-172) is increased as compared to a control tumor (27-165). B. *Zfp521* expression levels in the same AKXD27 tumors. C. *cyclin D1* expression levels in leukemia cell lines. The NFS-467 cell line has a retroviral insertion at the *Evi3* locus, and shows a significant increase in *cyclin D1* expression as compared to BCL1 cells transfected with the *Zfp521* scrambled shRNA (*p < 0.05; *t* test). D. The expression levels of *cyclin D1* in BCL1 cells transfected with either the *Zfp521* shRNA or scrambled shRNA at 24 and 48 h post-transfection. A decrease in *cyclin D1* expression is shown in *Zfp521* shRNA transfections compared to cells transfected with scrambled shRNA sequence (*p < 0.1; *t* test).

Based on the finding that *cyclin D1* rescued cell viability in *Zfp521* knockdown cells, we examined whether AKXD27 B-cell tumors had perturbed expression of *cyclin D1*. We found that *cyclin D1* expression is up regulated in tumors from AKXD27 mice with retroviral insertions at *Evi3* as compared to a control tumor without an *Evi3* retroviral insertion (Fig. 5A). *Zfp521* over-expression is also detected in the tumors with retroviral insertions at the *Evi3* locus (Fig. 5B). Furthermore, *cyclin D1* expression is increased in a B-cell leukemia cell line (NSF-467) with a retroviral insertion at the *Evi3* locus, when compared to B-cell lines without *Evi3* retroviral insertions (Fig. 5C). We also confirmed that *cyclin D1* expression is reduced in cells with *Zfp521* knockdown compared to control cells, indicating that *cyclin D1* is downstream of *Zfp521* in B-cells (Fig. 5D).

## 4 Discussion

*Zfp521* encodes a transcription factor with an N-terminal transcriptional repressor motif [30] and 30 Kruppel-like-zinc finger domains [8], [9]. Due to the increased numbers of B-cells in mice with *Zfp521* over-expression, we postulated that *Zfp521* may function during B-cell development to regulate B-cell viability or proliferation. Additionally, the finding that mice with *Zfp521* over-expression have an inappropriate B-cell surface marker profile, with upregulation of markers expressed from the pro-B stage of differentiation [8], raises the possibility that *Zfp521* functions as part of the B-cell transcription factor network to promote differentiation events.

The results from our *Zfp521* knockdown culture system are consistent with the above hypotheses. We discovered that BCL1 cell viability is reduced and apoptosis is significantly increased when *Zfp521* is knocked down, suggesting a role for *Zfp521* in B-cell survival. Our results support a role for *Zfp521* in the regulation of genes expressed from the Pro-B-stage onwards. These results are consistent with the findings from a recent study reporting a role for *Zfp521* in regulation of the pre-B-cell receptor genes *BTK*, *BANK1*, and *BLNK* [14]. Moderate levels of *ZNF521* expression have also been noted in the human Raji (pre-B) cell line [10].

Retroviral insertion at the *Evi3* locus causing *Zfp521* over-expression is a cooperative event in the development of acute B-lineage leukemia in mice expressing an E2A-HLF chimeric protein associated with acute lymphoblastic leukemia t(17;19) [31]. The tumors in these mice are of the B-progenitor type, and express B220 and Cd19, similar to tumors found in AKXD27 mice with retroviral insertions at the *Evi3* locus [8]. Further studies revealed *ZNF521* overexpression in human leukemic cell lines with t(17;19) translocations [31], suggesting that the mechanism by which over-expression of *Zfp521/ZNF521* causes B-cell leukemia may be conserved between mouse and human. Transgenic mice that over-express both the *E2A-HLF* fusion gene and *Zfp521* showed increased proliferation of B-cell progenitors [31]. Our study suggests that the increased proliferation found in *Zpf521* over-expressing Pro-B-cells is mediated by *cyclin D1*. Additionally, *Zfp521* has been reported to regulate *cyclin D3* expression in Pre-B-cells [14], suggesting that perturbations of *Zfp521* expression disrupt the G1/S transition in the cell cycle.

The knockdown of *Zfp521* in cultured hematopoietic progenitor cells has been reported to increase B-cell differentiation, as well as increase overall cell number in the culture [30]. The B-cell genes *Ebf1*, *Pax5*, and *Mb1* were also up-regulated in these knockdown cultures [30]. It is possible that knocking down *Zfp521* at the Pro-B-cell stage has an opposite effect to its knockdown in progenitor cells. Knockdown of *Zfp521* at the Pre-B-cell stage also reduces cell proliferation [14], similar to our findings at the Pro-B-cell stage. Notably, in Pro-B-cell tumours with *Evi3* retroviral insertions, *Zfp521* over-expression occurs concomittantly with *Ebf1*, *Pax5*, and *Mb1* over-expression, suggesting that *Zfp521* has a positive effect on the transcription of these B-cell factors at the Pro-B-cell stage [8]. We therefore propose that Zfp521 functions within the B-cell transcriptional network to promote differentiation events subsequent to the Pro-B-cell stage of lineage commitment. Further study is needed to determine how perturbations of Zfp521 function at different stages of B-cell development disrupt the balance of gene expression patterns and cellular activities to culminate in leukemic transformation of B-lineage cells.

## Conflict of interest

The authors declare that there are no conflicts of interest.
