# Supplementary material for: Zfp521 promotes B-cell viability and cyclin D1 gene expression in a B cell culture system
Source: Leuk Res. 2016 Jul;46:10–7. doi: 10.1016/j.leukres.2016.03.013 (PMC4910839; doi:10.1016/j.leukres.2016.03.013)
Supplement: Supplementary file 1 [file mmc1.docx]

Supplemental table 1

| **Gene** | **Forward Primer (5’-3’)** | **Reverse Primer (5’-3’)** |
| --- | --- | --- |
| ***18s*** | cca-tcc-aat-cgg-tag-tag-cg | gta-acc-cgt-tga-acc-cca-tt |
| ***Zfp521*** | gcc-ctg-gac-tgc-ctt-acc | tgt-cac-tgt-ggc-tct-gtt-cat |
| ***Zfp423*** | ctc-ttg-act-tgt-cac-gct-gtt | aac-agc-gtg-aca-agt-caa-gag |
| ***Runx1*** | cac-cga-cag-ccc-caa-ct | ccc-cag-tgc-cac-cac-ct |
| ***Lrf*** | gtc-gca-gaa-ggt-gga-gaa-gaa-gat | agc-cgt-ctt-tct-tga-ggt-gtc-tct |
| ***Ebf1*** | tgg-aca-act-ggc-tgt-gaa-tgt-c | acc-aat-caa-ggt-ttc-acc-cg |
| ***E2f2*** | tga-atg-ccg-agc-aga-cct-tgg-a | ctt-gac-cgc-aat-cac-tgt-ctg-c |
| ***Rag1*** | ggc-tag-ggt-cag-cag-caa-gga | gaa-cac-att-ctg-gct-gat-ccc-gtg |
| ***Ikaros*** | gct-ggc-tct-caa-gga-gga-g | gct-gaa-ggt-gta-caa-gtg-cg |
| ***Cyclin D1*** | cac-acg-gac-tac-agg-gga-gt | cac-agg-agc-tgg-tgt-tcc-at |
| ***Pax5*** | aac-cca-tca-agc-cag-aac-ag | ggg-gaa-cct-cca-aga-atc-at |

*Zfp521* primers were designed and purchased from Primer Design. All others were purchased from Eurogentec.
